# Association of *IL-12B rs3212227* and *IL-6 rs1800795 *Polymorphisms with Susceptibility to Cervical Cancer: A Systematic Review and Meta-Analysis

**DOI:** 10.31557/APJCP.2020.21.5.1197

**Published:** 2020-05

**Authors:** Mojgan Karimi-Zarchi, Hajar Abbasi, Atiyeh Javaheri, Amaneh Hadadan, Bahare Meibod, Razieh Sadat Tabatabaei, Yaser Ghelmani, Hossein Neamatzadeh

**Affiliations:** 1 *Department of Obstetrics and Gynecology, Iran University of Medical Sciences, Tehran, Iran. *; 2 *Endometriosis Research Center, Iran University of Medical Sciences, Tehran, Iran. *; 3 *Department of Obstetrics and Gynecology, Shahid Beheshti University of Medical Sciences, Tehran, Iran. *; 4 *Department of Obstetrics and Gynecology, Shahid Sadoughi University of Medical Sciences, Yazd, Iran. *; 5 *Department of Internal Medicine, Shahid Sadoughi University of Medical Sciences, Yazd, Iran. *; 6 *Clinical Research Development Center of Shahid Sadoughi Hospital, Shahid Sadoughi University of Medical Sciences, Yazd, Iran. *; 7 *Department of Medical Genetics, Shahid Sadoughi University of Medical Sciences, Yazd, Iran. *; 8 *Mother and Newborn Health Research Center, Shahid Sadoughi University of Medical Sciences, Yazd, Iran. *

**Keywords:** Cervical cancer, cervix, interleukin, polymorphism, association, meta-analysis

## Abstract

**Background:**

Primary studies have shown that the *IL-12B rs3212227* and *IL-6 rs1800795 *polymorphisms are associated with an increased risk of cervical cancer. However, conflicting results warrant a meta-analysis to obtain more precise estimates.

**Methods:**

A comprehensive literate search on PubMed, Web of Science, Scopus, CNKI, and SciELO was performed to collect all eligible studies up to November 10, 2019. The pooled odds ratios (OR) and 95% confidence intervals (CI) were used to calculate the risk. This meta-analysis was carried out by utilizing CMA software.

**Results:**

A total of eleven case-control studies including four studies on *IL-12B rs3212227* and seven studies on *IL-6* *rs1800795* were selected. Pooled ORs revealed that the *IL-6 rs1800795* polymorphism was significantly associated with an increased risk of cervical cancer (C vs. G: OR = 1.294, 95% CI 1.071-1.564, p= 0.007; CC vs. GG: OR = 1.633, 95% CI 1.059-2.520, p= 0.027; CC+CG vs. GG: OR = 1.312, 95% CI 1.048-1.643, p= 0.018; and CC vs. CG+GG: OR = 1.592, 95% CI 1.268-1.999, p≤0.001), but not *IL-12B rs3212227* polymorphism. Stratified analysis by ethnicity revealed that both *IL-12B rs3212227* and *IL-6 rs1800795* polymorphisms were associated with risk of cervical cancer in Asian women.

**Conclusions:**

Our pooled data revealed that the* IL-12B rs3212227 *and *IL-6 rs1800795* polymorphisms may be used to identify individuals at high risk of cervical cancer in Asian women.

## Introduction

Cervical cancer is the second most commonly diagnosed malignancy and third leading cause of cancer-related mortality among women in less developed countries (Karimi Zarchi et al., 2010; Mousavi et al., 2008). According to GLOBOCAN, cervical cancer accounts for 528,000 new cases and 266,000 deaths worldwide in 2012 (Parkin et al., 2005). Although, it has been widely accepted for several decades that Human papillomavirus (HPV) is the main risk factor for development of cervical cancer (Behtash et al., 2009; Farbod et al., 2019; Ghaemmaghami et al., 2008), HPV infections have developed to persistent infection in only a very few cases and it is sufficient to induce cervical cancer in an even smaller proportion (Cheng et al., 2018). Thus, many scientists acknowledge that etiology of cervical cancer is multifactorial and several factors such as sexually transmitted infections, reproductive factors, hormonal influences, genetics and host factors are responsible for development of cervical cancer (Momenimovahed and Salehiniya, 2017; Akhavan et al., 2012). However, studies revealed a preventive role for antiretroviral therapy (ART) on cervical lesion incidence and progression and promotion of regression (de Vries et al., 2018; Jarahzadeh et al., 2014; Jarahzadeh et al., 2011). In the recent years, several number of genome-wide association studies (GWAS) have been performed to evaluate the effects of different cytokines in development of cervical cancer, and many case-control studies have reported the association of *IL-12* and *IL-6 *with an increased risk of cervical cancer.

*IL-12* is a potent pro-inflammatory cytokine that plays a central role in cellular immunity which is mainly produced by phagocytic cells, such as dendritic cells, monocytes, macrophages, neutrophils, and microglia in response to antigenic stimulation. *IL-12* has a number of proinflammatory biological functions and its pleiotropic functions which drive cellular immunity support its exploration as an antitumor agent. Therefore, in clinical studies it has been evaluated as an experimental agent for treatment of numerous tumours (Van Herpen et al., 2005; Xu et al., 2004). The *IL-12B* gene is mapped to chromosome 5q31-33 and encodes the p40 subunit of interleukin 12 (Randolph et al., 2004). Moreover,* IL-6 *is a multipotent cytokine that regulates immune and inflammatory responses (Aflatoonian et al., 2019b; Salimi et al., 2019; Sheikhpour et al., 2017). Moreover, *IL-6* has been considered as a “metabolic hormone” which affects metabolism of glucose, protein and lipids (Gholami et al., 2019). The human *IL-6* gene is localized to chromosome 7p21-24, consists of seven exons and spanning 12.8 kb (Salimi et al., 2019).

Up to now, many related articles have evaluated the association of *IL-12 rs3212227* and *IL-6 rs1800795 *polymorphisms with risk of cervical cancer. Some epidemiological studies suggested that *IL-12B rs3212227 *and *IL-6 rs1800795* polymorphisms are associated with an increased risk of cervical cancer. However, two recent meta-analyses were performed to evaluate *IL-12B rs3212227* and *IL-6 rs1800795* polymorphisms with cervical cancer risk and their results indicated that no significant association was found among all ethnicities (Chang et al., 2015; Liu et al., 2017; Pu et al., 2016; Zheng et al., 2017). Therefore, controversy and inconformity still remained about *IL-12B rs3212227 *and *IL-6 rs1800795* polymorphisms and most of studies recruited a limited sample capacity, which is insufficient to get a firm conclusion about the associations of these polymorphisms with development of cervical cancer. Thus, we systematically reviewed previous publications and a meta-analysis was conducted to evaluate the associations of *IL-12B rs3212227* and *IL-6 rs1800795* polymorphisms with risk of cervical cancer.

## Materials and Methods

*Primary Search Strategy*

Ethical approval was not necessary for the present meta-analysis. We systematically searched PubMed, EMBASE, Wed of Science, Elsevier, Google Scholar, Cochrane Library, SciELO, SID, WanFang, VIP, Chinese Biomedical Database (CBD) and Chinese National Knowledge Infrastructure (CNKI) comprehensively for all publications regarding the association *IL-12B rs3212227* and* IL-6 rs1800795* polymorphisms and cervical cancer risk up to November 10, 2019. The combination of following keywords and terms were used: (‘’Uterine Cervical Neoplasm‘’ OR ‘’Cervix Cancer’’ OR ‘’Cervical Cancer’’ OR ‘’Cervical Neoplasm’’ OR ‘’Cervical Carcinoma’’) AND (‘’Interleukin 12’’ OR ‘’IL-12’’ OR ‘’1188A>C’’ OR ‘’rs3212227’’) AND (‘Interleukin 6’’ OR ‘’ -174G>C’’ OR ‘’rs1800795’’) AND (‘‘Polymorphism’’ OR ‘‘Mutation’’ OR ‘‘Genotype’’ OR ‘‘Allele’’ OR ‘‘Variation’’ OR ‘‘Variant’’). Meanwhile, hand searching of the references in retrieved reviews and eligible articles were performed as sources to find other relevant publications. Languages were limited to English, Portuguese, Farsi and Chinese.

*Including and Excluding Criteria*

We set these inclusive criteria for recruited publications: a) studies with case-control or cohort design; 2) published studies in English, Chinese and Farsi; 3) studies evaluated the association of *IL-12B rs3212227* and *IL-6 rs1800795 *polymorphisms with risk of cervical cancer; and 4) enough and available data to figure out odds ratios (ORs) and 95% confidence intervals (CIs). In addition, we also restricted these exclusive criteria: 1) studies without control group (case only studies); 2) insufficient data offered to analyze or unavailable data; 3) studies were carried out based on animals and in vitro studies; 4) studies evaluated the association of other polymorphisms at *IL-12* and* IL-6 *genes with cervical cancer; 5) case reports, case series, letters, comments, reviews, and previous meta-analyses 6) overlapped data or duplication.

*Data extraction*

All recruited studies had to be seriously scanned by two individual researchers separately. If there was a dispute between the two researchers, they would reach a consensus by discussing or a third researcher. We extracted the following information from eligible studies: name of the first author, year of publication, country of origin, ethnicity of participants, genotyping methods, source of controls, total numbers of cases and controls, genotyping method, genotypes frequencies of cases and controls, minor allele frequencies (MAFs) and Hardy-Weinberg equilibrium test in control subjects.

*Statistical Analysis*

An ethical approval was not necessary as this study was a meta-analysis based on previous studies. The strength of association of *IL-12B rs3212227* and *IL-6 rs1800795* polymorphisms with risk of cervical cancer was measured by odds ratios (ORs) with 95% confidence intervals (CIs). The statistical significance of the pooled OR was determined using the Z-test. Pooled estimates of the OR were obtained by calculating a weighted average of OR from each study. The pooled ORs was calculated under all five genetic models, i.e., allele (B vs. A), homozygote (BB vs. AA), heterozygote (BA vs. AA), dominant (BB+BA vs. AA) and recessive (BB vs. BA+AA). A* χ*^2^-based Q test was calculated for assessing the heterogeneity among recruited investigations and if the P-value of Q test exceeded 0.05 that meant there was no obvious heterogeneity. In addition, I^2^-value was used to quantify the proportion of the between study heterogeneity (range of 0 to 100%: I^2^=0-25%, no heterogeneity; I^2^=25-50%, moderate heterogeneity; I^2^= 50–75%, large heterogeneity; I^2^=75–100%, extreme heterogeneity). Random-effect models (DerSimonian-Laird method) would be adopted for analyses if I^2^ was >50%. Otherwise, analyses would be conducted with fixed-effect models (Mantel- Haenszel method). Genotype frequencies of controls for each study using goodness-of-fit test (chi-square) and a p-value less than 0.05 was considered as significant disequilibrium (HWE-violating). Sensitivity analysis was conducted by excluding one study at a time to examine the stability of the pooled results. Begg’s funnel plot and Egger’s linear regression test were applied to assess potential publication bias, in which P<0.05 was considered statistically significant. All of the statistical calculations were performed using Comprehensive Meta-Analysis (CMA) software version 2.0 (Biostat, USA). Two-sided P-values < 0.05 were considered statistically significant.

## Results

*Characteristics of Included Studies*

By electronic and manual searches concerning the association of *IL-12B rs3212227* and *IL-6 rs1800795 *polymorphisms and cervical cancer risk, 297 relevant studies up to November 05, 2019 were identified. After reading titles and abstracts, 139 irrelevant and duplicate articles were excluded. Another 95 articles were subsequently excluded because not reporting useful data for meta-analysis, review, case only study, and not being case-control studies. Finally, a total of eleven case-control studies including four studies on *IL-12B rs3212227* (Chen et al., 2009; Do Carmo Vasconcelos De Carvalho et al., 2012; Han et al., 2008; Roszak et al., 2012) and seven studies on *IL-6 rs1800795 *(Gangwar et al., 2009; Grimm et al., 2011; Nogueira De Souza et al., 2006; Pu et al., 2016; Shi et al., 2013; Zidi et al., 2017) were selected. Characteristics of included studies are shown in Table 1. All eligible studies were published in English and Chinese between April, 2008 and November, 2017. Among them, five studies were based on Asians, three studies based on mixed populations, two studies based on Caucasians, and one study was based on African populations. The selected studies were conducted in Korea, China, Brazil, Poland, India, Austria and Tunisia. Five different genotyping methods including SNaPShot, PCR-RFLP, direct Sequencing, TaqMan and qRT-PCR were used by selected studies. The allele, genotype and minor allele frequency (MAF) distributions in the cases and controls are shown in Table 1. Moreover, the distribution of genotypes in the controls was in agreement with Hardy-Weinberg equilibrium (HWE) for all selected studies, except for two studies on IL-6 rs1800795 polymorphism (Table 1).

*Quantitative Data Synthesis*

*IL-12B rs3212227 Polymorphism*

The summary of the meta-analysis of the association of between *IL-12B rs3212227* polymorphism and cervical cancer risk are shown in Table 2. Overall, there was no significant association between *IL-12B rs3212227 *polymorphism and cervical cancer risk under all five genetic models genetic models, i.e., allele (C vs. A: OR = 1.076, 95% CI 0.783-1.479, p=0.651, Figure 2A), homozygote (CC vs. AA: OR = 1.330, 95% CI 0.988-1.790, p=0.060), heterozygote (CA vs. AA: OR = 1.119, 95% CI 0.696-1.800, p=0.643), dominant (CC+CA vs. AA: OR = 1.125, 95% CI 0.704-1.799, p =0.623, Fig2B), recessive (CC vs. CA+AA: OR = 1.140, 95% CI 0.874-1.487, p =0.333). However, subgroup analysis by ethnicity showed a significant association between IL-12B rs3212227 polymorphism and an increased risk of cervical cancer in Asian women (CA vs. AA: OR = 1.349, 95% CI 1.032-1.762, p=0.028 and CC+CA vs. AA: OR = 1.340, 95% CI 1.041-1.725, p =0.023, Table 2).

*IL-6 rs1800795 Polymorphism*

The summary of the meta-analysis of the association of between *IL-6 rs1800795* polymorphism and cervical cancer risk are shown in Table 2. Pooled ORs revealed that *IL-6 rs1800795* polymorphism was significantly associated with an increased risk of cervical cancer under four genetic models, i.e., allele (C vs. G: OR = 1.294, 95% CI 1.071-1.564, p= 0.007, Figure 2C), homozygote (CC vs. GG: OR = 1.633, 95% CI 1.059-2.520, p= 0.027), dominant (CC+CG vs. GG: OR = 1.312, 95% CI 1.048-1.643, p= 0.018) and recessive (CC vs. CG+GG: OR = 1.592, 95% CI 1.268-1.999, p≤0.001, Figure 2D). Moreover, subgroup analysis by ethnicity revealed an increased risk of cervical cancer in Asian women. When stratified by country of origin, there was a significant association between *IL-6 rs1800795* polymorphism and cervical cancer risk among Chinese women (C vs. G: OR = 1.422, 95% CI 1.025-1.971, p= 0.035; CC vs. GG: OR = 1.851, 95% CI 1.377-2.488, p≤0.001; and CC vs. CG+GG: OR = 1.641, 95% CI 1.251-2.151, p≤0.001), but not among Brazilian women (Table 2).

*Between-Study Heterogeneity Test*

As shown in Table 2, there was significant between-study heterogeneity under almost genetic models for both *IL-12B rs3212227 *and *IL-6 rs1800795* polymorphisms in the overall population. To explore the potential sources of heterogeneity, subgroup analyses by ethnicity, country of origin and HWE was performed. The results suggested that ethnicity might be contributed to between-study heterogeneity in the current meta-analysis.

*Sensitivity Analysis*

A sensitivity analysis was used to test the effects of each study on pooled ORs. There were no significant differences observed upon removal of any of the studies, suggesting that our findings were statistically robust and reliable. Moreover, we performed sensitivity analysis by excluding those HWE-violating studies for IL-6 rs1800795. However, after excluding those studies the pooled ORs were not changed statically in the overall population, indicating that this meta-analysis result were statistically robust and reliable.

*Publication Bias*

Begg’s funnel plot and Egger’s test were inspected to evaluate the possible publication bias in this meta-analysis. The shape of the funnel did not show any obvious asymmetry under all five genetic models for both* IL-12B rs3212227* and *IL-6 rs1800795 *polymorphisms (Figure 3). Moreover, Egger’s test was statistically revealed that there was no a significant bias for IL-12B rs3212227 polymorphism (C vs. A: P_Beggs_ = 0.308; P_Eggers_ = 0.236; CC vs. AA: PBeggs = 0.734, PEggers = 0.782; CA vs. AA: P_Beggs_ = 0.308, P_Eggers_ = 0.356; CC+CA vs. AA: P_Beggs_ = 0.308, P_Eggers_ = 0.317; and CC vs. CA+AA: P_Beggs_ = 0.734, P_Eggers_ = 0.946) and IL-6 rs1800795 polymorphism (C vs. G: P_Beggs_ = 0.763, P_Eggers_ = 0.701; CC vs. GG: PBeggs = 0.763, P_Eggers_ = 0.587; CG vs. GG: P_Beggs_ = 0.763, P_Eggers_ = 0.728; CC+CG vs. GG: P_Beggs_ = 1.000, PEggers = 0.583; and CC vs. CG+GG: P_Beggs _= 0.548, P_Eggers_ = 0.646).

**Figure 1:**
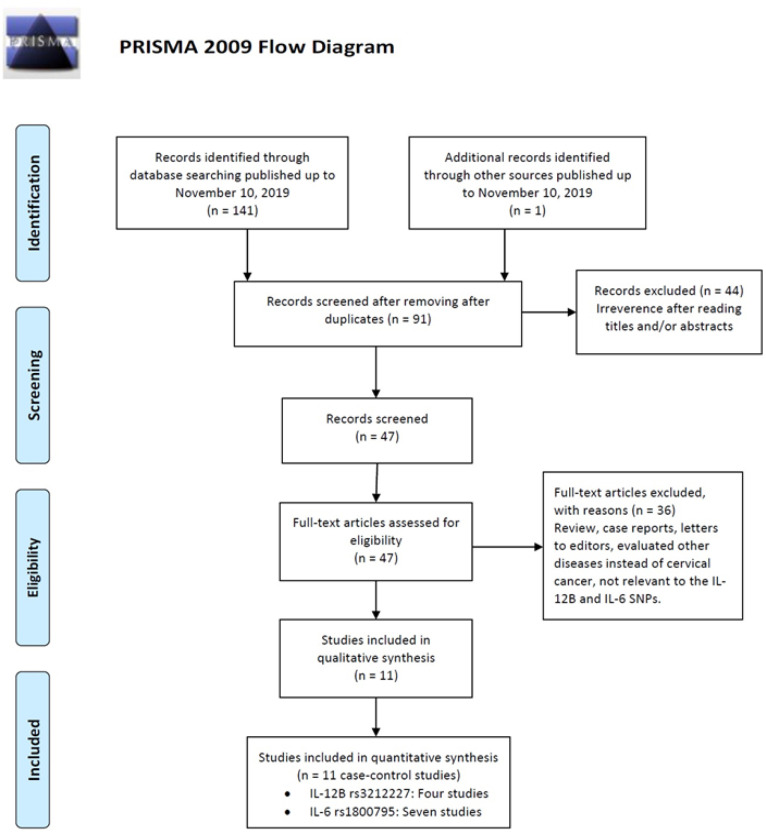
Flow Chart for the Process of Selecting Eligible Studies

**Table 1:** Characteristics of All Studies Included in the Current Meta-Analysis

First Author	Country	Genotyping	SOC	Case/Control	Cases					Controls					MAFs	HWE
	(Ethnicity)	Methods			Genotypes			Allele		Genotypes			Allele			
IL-12B rs3212227					AA	AC	CC	A	C	AA	AC	CC	A	C		
Han 2008	Korea(Asian)	SNaPShot	HB	150/179	32	87	31	151	149	52	88	39	192	166	0.464	0.877
Chen 2009	China(Asian)	PCR-RFLP	PB	404/404	127	199	78	453	355	150	185	69	485	323	0.4	0.357
de Carvalho 2012	Brazil(Mixed)	PCR-RFLP	PB	162/76	100	49	13	249	75	31	37	8	99	53	0.349	0.531
Roszak 2012	Poland(Caucasian)	PCR-RFLP	PB	405/450	212	174	19	598	212	289	151	10	729	171	0.19	0.055
IL-6 rs1800795					GG	GC	CC	G	C	GG	GC	CC	G	C		
de Souza 2006	Brazil(Mixed)	PCR-RFLP	PB	56/253	24	32	0	80	32	148	102	3	398	108	0.213	0.001
Gangwar 2009	India(Asian)	ARMS-PCR	HB	160/200	107	36	17	250	70	142	51	7	335	65	0.163	0.371
Grimm 2011	Austria(Caucasian)	Sequencing	HB	131/208	55	51	25	161	101	85	96	27	266	150	0.361	0.989
Junior 2012	Brazil(Mixed)	Sequencing	PB	115/115	72	39	4	183	47	67	37	11	171	59	0.257	0.093
Shi 2014	China(Asian)	PCR-RFLP	HB	518/518	160	253	105	573	463	181	259	78	621	415	0.401	0.348
Pu 2016	China(Asian)	TaqMan	HB	360/728	185	141	34	511	209	476	220	32	1172	284	0.195	0.309
Zidi 2017	Tunisia(African)	qRT-PCR	HB	112/164	81	25	6	187	37	133	25	6	291	37	0.113	0.002

PCR, polymerase chain reaction; RFLP, restriction fragment length polymorphism; ARMS, amplification-refractory mutation system; qRT-PCR, Real-Time qRT-PCR; SOC, source of controls; HB, hospital-based; PB, population-based; MAFs, minor allele frequencies; HWE, Hardy-Weinberg equilibrium

**Table 2:** Summary Risk Estimates for Association of IL-12B rs3212227 and IL-6 rs1800795 Polymorphisms with Cervical Cancer Risk

Subgroup	Genetic Model	Type of Model	Heterogeneity		Odds Ratio				Publication Bias	
			I^2^ (%)	P_H_	OR	95% CI	Z_test_	P_OR_	P_Beggs_	P_Eggers_
*IL-12B rs3212227*										
Overall Population	C vs. A	Random	81.69	0.001	1.076	0.783-1.479	0.452	0.651	0.308	0.236
	CC vs. AA	Random	54.75	0.085	1.33	0.988-1.790	1.878	0.06	0.734	0.782
	CA vs. AA	Random	82.52	0.001	1.119	0.696-1.800	0.463	0.643	0.308	0.356
	CC+CA vs. AA	Random	83.68	≤0.001	1.125	0.704-1.799	0.492	0.623	0.308	0.317
	CC vs. CA+AA	Random	24.76	0.263	1.14	0.874-1.487	0.969	0.333	0.734	0.946
Asians	C vs. A	Fixed	0	0.87	1.166	0.988-1.377	1.812	0.07	NA	NA
	CC vs. AA	Fixed	0	0.932	1.323	0.941-1.860	1.61	0.107	NA	NA
	CA vs. AA	Fixed	0	0.454	1.349	1.032-1.762	2.191	0.028	NA	NA
	CC+CA vs. AA	Fixed	0	0.594	1.34	1.041-1.725	2.271	0.023	NA	NA
	CC vs. CA+AA	Fixed	0	0.507	1.086	0.807-1.461	0.542	0.588	NA	NA
*IL-6 rs1800795 *										
Overall Population	C vs. G	Random	60	0.02	1.294	1.071-1.564	2.675	0.007	0.763	0.701
	CC vs. GG	Random	54.07	0.042	1.633	1.059-2.520	2.217	0.027	0.763	0.587
	CG vs. GG	Random	52.47	0.049	1.232	0.971-1.562	1.718	0.086	0.763	0.728
	CC+CG vs. GG	Random	53.01	0.047	1.312	1.048-1.643	2.371	0.018	1	0.583
	CC vs. CG+GG	Fixed	48.97	0.068	1.592	1.268-1.999	3.999	≤0.001	0.548	0.646
Asians	C vs. G	Fixed	65.97	0.053	1.395	1.230-1.582	5.195	≤0.001	1	0.793
	CC vs. GG	Fixed	56.74	0.099	1.951	1.472-2.584	4.656	≤0.001	1	0.375
	CG vs. GG	Fixed	66.54	0.05	1.289	1.077-1.543	2.769	0.006	1	0.613
	CC+CG vs. GG	Fixed	61	0.077	1.429	1.207-1.692	4.136	≤0.001	1	0.766
	CC vs. CG+GG	Fixed	53.87	0.114	1.736	1.339-2.251	4.166	≤0.001	0.296	0.205
By Country										
China	C vs. G	Random	82.88	0.016	1.422	1.025-1.971	2.111	0.035	NA	NA
	CC vs. GG	Fixed	70.12	0.067	1.851	1.377-2.488	4.083	≤0.001	NA	NA
	CG vs. GG	Random	75.83	0.042	1.351	0.912-1.999	1.502	0.133	NA	NA
	CC+CG vs. GG	Random	77.92	0.033	1.466	0.994-2.162	1.929	0.054	NA	NA
	CC vs. CG+GG	Fixed	56.13	0.131	1.641	1.251-2.151	3.581	≤0.001	NA	NA
Brazil	C vs. G	Random	77.49	0.035	1.043	0.534-2.307	0.123	0.902	NA	NA
	CC vs. GG	Fixed	0	0.568	0.385	0.127-1.164	-1.691	0.091	NA	NA
	CG vs. GG	Fixed	62.94	0.1	1.356	0.905-2.032	1.474	0.14	NA	NA
	CC+CG vs. GG	Fixed	75.47	0.043	1.239	0.559-2.748	0.528	0.598	NA	NA
	CC vs. CG+GG	Fixed	0	0.704	0.37	0.124-1.105	-1.781	0.075	NA	NA
HWE*	C vs. G	Random	71.99	0.006	1.239	0.979-1.564	1.783	0.075	0.806	0.451
	CC vs. GG	Random	68.89	0.012	1.633	0.978-2.727	1.876	0.061	0.806	0.682
	CG vs. GG	Fixed	57.83	0.05	1.198	1.019-1.407	2.194	0.028	0.462	0.18
	CC+CG vs. GG	Random	63.28	0.028	1.213	0.928-1.587	1.412	0.158	0.22	0.163
	CC vs. CG+GG	Random	64.82	0.023	1.588	1.004-2.510	1.977	0.048	1	0.827

NA, Not Applicable; *, By excluding HWE-violating studies

**Figure 2:**
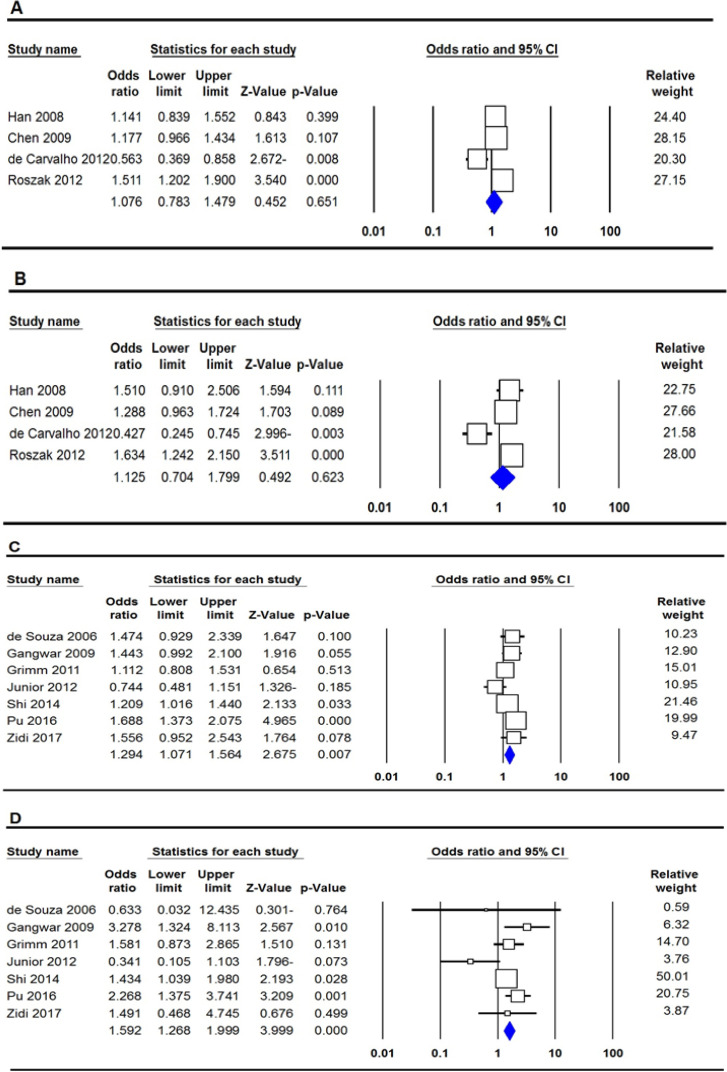
Forest Plot for the Association of *IL-12B rs3212227* and *IL-6 rs1800795* Polymorphisms with Risk of Cervical Cancer in the Overall Population. A, *IL-12B rs3212227* (allele model: C vs. A); B, *IL-12B rs3212227* (dominant model: CC+CA vs. AA); C, *IL-6 rs1800795* (allele model: C vs. G); D, IL-6 rs1800795 (recessive model: CC vs. CG+GG)

**Figure 3:**
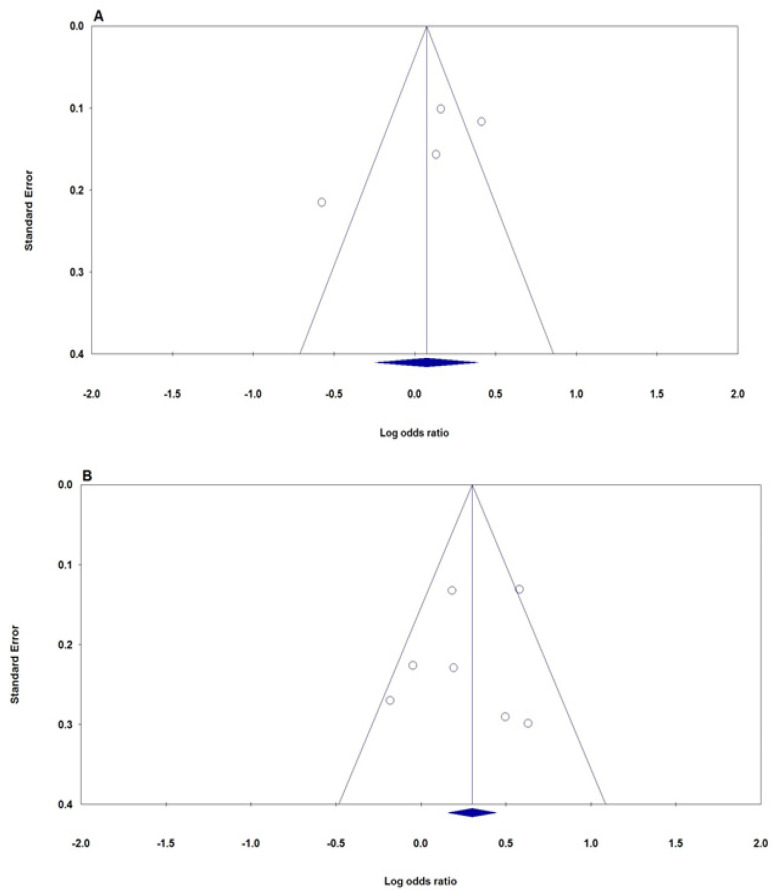
Begg’s Funnel Plot for the Association of *IL-12B rs3212227* and *IL-6 rs1800795* Polymorphisms with Risk of Cervical Cancer. A, *IL-12B rs3212227* (allele model: C vs. A); B, *IL-6 rs1800795* (dominant model: CC+CG vs. GG)

## Discussion

Our pooled data showed that *IL-12B rs3212227 *polymorphism was not significantly associated with an increased risk of cervical cancer. However, our subgroup analysis revealed that *IL-12B rs3212227 *polymorphism was significantly associated with risk of cervical cancer in Asians. Roszak et al., in a case-control study reported that the *IL-12B rs3212227* might be contributed to an increased risk of cervical cancer in Polish population. However, de Carvalho et al., (2012) reported that *IL-12B rs3212227* polymorphism has a protective effect in development of cervical cancer among Brazil women. Moreover, Han et al., (2008) in an earlier study identified that there was no relationship between cervical cancer and *IL-12B rs3212227* in Korean population. In 2017, Zheng et al., (2017) in a meta-analysis of 33 studies with 10,587 cancer cases and 12,040 cancer-free controls evaluated association of *IL-12B rs3212227* with cancer risk. Their pooled data showed a significant association between *IL-12B rs3212227* polymorphisms and overall cancer risk, especially in hepatocellular carcinoma, nasopharyngeal cancer, and among Asians. However, their stratification analyses demonstrated that* IL-12B rs3212227* was not associated with cervical cancer. In 2015, Chang et al., (2015) in a meta-analysis of five case-control studies with 2,552 cases and 2,232 controls were evaluated association of *IL-12B rs3212227* with cervical cancer. Their results revealed that *rs3212227* polymorphism in *IL-12* gene may not be the risk factor to cervical cancer.

In 2006, Nogueira De Souza et al., (2006) for first time in a case-control study with 56 cases and 253 controls evaluated the association of IL-6 rs1800795 polymorphism with cervical cancer in a Brazilian population. Since a few studies evaluated the association in different population. Our pooled data revealed that *IL-6 rs1800795* polymorphism was associated with an increased risk of cervical cancer in the overall population. Similarly, Duan et al., (2018) in a meta-analysis of seven publications found that *IL-6 rs1800795* polymorphism was associated with cervical cancer risk. In 2017, Zidi et al., (2017) in a case-control study evaluated the effects of* IL-6 rs2069845*, *rs2069840*, *rs1474348*,* rs1800795*, *rs1800797*, and *rs2069827* polymorphisms with risk of cervical cancer among Tunisian women. Their results revealed high linkage disequilibrium (LD) between *rs2069845*, *rs2069840*,* rs1474348*, and *rs1800795 *polymorphism. Moreover, their 6-locus haplotype analysis have identified that GACCCA haplotype to be positively associated with an increased risk of cervical cancer, while GAGGGG haplotype was negatively associated with occurrence of cervical cancer, thus suggesting a protective role for this haplotype in development of this disease. They have reported that *IL-6 rs1800795* polymorphism might be has a protective role in development of cervical cancer. In 2016, Pu et al., (2016) in a case-control study of 360 cervical cancer cases and 728 controls evaluated the relationship between *rs1800795* and* rs2069837* polymorphisms at *IL-6* and cervical cancer among Chinese women. Their results revealed that mutant alleles of *rs1800795* and *rs2069837 *polymorphisms were associated with an increased risk cervical cancer. de Lima et al., (2016) revealed that there was no significant association between the *IL-6 rs1800795* polymorphism and HPV infected women or uninfected controls. Moreover, they have not found any significant association between *TGF-β1 +869T>C* and *+915G>C *polymorphisms and the risk of HPV infection in the patients. Thus, they have hypothesized that the* IL-6 rs1800795* polymorphism did not play a role in the risk of HPV infection in the Brazilian population .

Between-study Heterogeneity in a meta-analysis refers to the variation in study outcomes between studies (Bahrami et al., 2019; Yazdi et al., 2017). Moreover, the extent to which effect sizes vary within a meta-analysis is called heterogeneity. Heterogeneity is to be expected in a meta-analysis and assessment of heterogeneity. Thus, the proportion of between-study heterogeneity is one of the major concerns in meta-analysis because nonhomogeneous study has high possibility to mislead results (Gohari et al., 2019). Therefore, the assessment of heterogeneity is necessary, as high heterogeneity could be caused by the fact that there are actually two or more subgroups of studies present in the data, which have a different true effect. According to our previous meta-analyses, we suggested that heterogeneity might be due to differences in study design, sample size, genotyping methods, disease heterogeneity, and to the different roles played by gene-gene and gene-environment interactions (Aflatoonian et al., 2019a; Mirjalili et al., 2018; Moghimi et al., 2019, 2018). Moreover, after subgroup an analysis, heterogeneity was obviously decreased in Asians; however, the corresponding pooled ORs were not materially altered after deleting these studies, indicating that our results were statistically robust. Publication bias is another key factor that might affect the quality of a meta-analysis (Niktabar et al., 2019; Veisian et al., 2019). Both Begg’s and Egger’s test were used to assess publication bias in this study. No significant publication bias was observed when all studies were included.

Several limitations should be acknowledged for interpretation of our pooled ORs. First, in the current meta-analysis we have selected only eligible published articles, so some relevant unpublished studies might be missed, generating certain publication bias, though not detected even with funnel plot or Egger test. Second, the sample size for both *IL-12B rs3212227* and *IL-6 rs1800795* polymorphisms was relatively small, with limited statistical power to explore the associations. Third, included studies are still so limited that we cannot perform subgroup analysis by ethnicity, genotyping methods and source of controls. Accordingly, it is required that a more precise analysis could be performed if sufficient individual study in globally populations were available. Fourth, there was heterogeneity between studies for both *IL-12B rs3212227* and *IL-6 rs1800795* polymorphisms which could distort our pooled data. Fifth, due to lack of original data in the selected studies restricted us to continue investigating some potential interactions, such as age, HPV infection, reproductive factors, hormonal influences, family history, environmental factors, and lifestyle. Hence, our pooled ORs might have some bias to a certain degree. Finally, cervical cancer is a complex disease with etiological factors that can be subdivided into extrinsic and intrinsic factors, whereas, in the current meta-analysis, the effects of these factors were not performed due to the insufficient data. Furthermore, plausible effects of gene-gene and gene-environment interactions on development of cervical cancer risk were not taken into account because of limited data as well.

In summary, our pooled data revealed that* IL-12B rs3212227* and *IL-6 rs1800795* polymorphisms could be used to identify individuals at high risk of cervical cancer in Asian women. In view of the above mentioned restrictions, high-quality researches with larger sample sizes are needed to assess the effect among different ethnicities and to validate our results.

## Competing of Interest

The authors do not have any conflict of interest to declare.
